# Solvent- and Light-Sensitive AIEE-Active Azo Dye: From Spherical to 1D and 2D Assemblies

**DOI:** 10.3390/ijms23020965

**Published:** 2022-01-16

**Authors:** Mina Han, Ikue Abe, Jihun Oh, Jaehoon Jung, Young Ji Son, Jaegeun Noh, Mitsuo Hara, Takahiro Seki

**Affiliations:** 1 Department of Chemistry Education, Kongju National University, Gongju 32588, Korea; 2 Department of Chemistry and Biotechnology, Graduate School of Engineering, Tottori University, Tottori 680-8552, Japan; hibari1116@gmail.com; 3 Department of Chemistry, University of Ulsan, Ulsan 44776, Korea; gminor231@gmail.com; 4 Department of Chemistry, Institute of Nano Science and Technology, Hanyang University, 222 Wangsimni-ro, Seongdong-gu, Seoul 04763, Korea; syj2010@hanyang.ac.kr (Y.J.S.); jgnoh@hanyang.ac.kr (J.N.); 5 Department of Molecular and Macromolecular Chemistry, Graduate School of Engineering, Nagoya University, Furo-cho, Chikusa-ku, Nagoya 464-8603, Japan; mhara@chembio.nagoya-u.ac.jp (M.H.); tseki@chembio.nagoya-u.ac.jp (T.S.)

**Keywords:** triangular azo dye, light and solvent response, molecular assembly, morphological transformation

## Abstract

Fluorescent molecular assembly systems provide an exciting platform for creating stimuli-responsive nano- and microstructured materials with optical, electronic, and sensing functions. To understand the relationship between (i) the plausible molecular structures preferentially adopted depending on the solvent polarity (such as N,N-dimethylformamide [DMF], tetrahydrofuran [THF], and toluene), (ii) the resulting spectroscopic features, and (iii) self-assembled nano-, micro-, and macrostructures, we chose a sterically crowded triangular azo dye (3Bu) composed of a polar molecular core and three peripheral biphenyl wings. The chromophore changed the solution color from yellow to pink-red depending on the solvent polarity. In a yellow DMF solution, a considerable amount of the twisted azo form could be kept stable with the help of favorable intermolecular interactions with the solvent molecules. By varying the concentration of the DMF solution, the morphology of self-assembled structures was transformed from nanoparticles to micrometer-sized one-dimensional (1D) structures such as sticks and fibers. In a pink-red toluene solution, the periphery of the central ring became more planar. The resulting significant amount of the keto-hydrazone tautomer grew into micro- and millimeter-sized 1D structures. Interestingly, when THF-H_2_O (1:1) mixtures were stored at a low temperature, elongated fibers were stacked sideways and eventually developed into anisotropic two-dimensional (2D) sheets. Notably, subsequent exposure of visible-light-irradiated sphere samples to solvent vapor resulted in reversible fluorescence off↔on switching accompanied by morphological restoration. These findings suggest that rational selection of organic dyes, solvents, and light is important for developing reusable fluorescent materials.

## 1. Introduction

Fluorescent molecular assembly systems provide an exciting platform for constructing reversibly stimuli-responsive assembled nano- and microstructures whose optical, electronic, and sensing functions can be regulated by contact and non-contact stimuli such as light, chemicals, temperature, and pressure [1,2,3,4,5,6,7,8,9,10,11,12,13,14,15,16,17,18,19,20,21,22,23,24,25]. Many studies have reported the self-assembly of π-conjugated organic chromophores into versatile fluorescent nano- and microstructured materials with such desired functions [1,2,3,4,5,6,12,14,21]. A sufficient understanding of (i) the design of small component molecules, (ii) specific and non-specific interactions of the molecules with surrounding solvents, and (iii) three-dimensional molecular arrangements is critical to elicit responses to stimuli from the self-assembled systems. For instance, the introduction of bulky substituents restricts the free rotation around a single or double bond, thus changing the twisting degree of the planar geometry. Such sterically crowded chromophores often undergo specific interactions with solvents with different polarities, and the resulting conformational and tautomeric structures may affect not only spectroscopic (absorption and emission) and sensing features but also molecular assemblies [26,27,28,29,30,31,32,33,34,35,36,37].

Azobenzene is a well-known photo-responsive compound, but is generally known to not emit with appreciable quantum yield (∼10^−7^–10^−5^) [38,39,40,41,42,43]. This is because the energy in the excited state is highly consumed through nonradiative conformational changes, thus reducing the fluorescence intensity significantly. However, Kawashima et al. developed intensely fluorescent boron-substituted azobenzenes [44,45,46]. The intramolecularly strong B–N interaction disrupts the molecular structural changes around the azo group, which is mainly responsible for the high fluorescence efficiency. Yoon et al. examined the aggregation-induced emission enhancement (AIEE [47,48,49,50,51,52]) property of *o*-phenylazonaphthol (*o*-PAN)-based chromophores [53]. The planarization associated with strong intramolecular hydrogen bonding and the restriction of intramolecular rotation in the hydrazone tautomer form are likely responsible for the enhanced fluorescence. Recently, Yamauchi and coworkers introduced a pyrene-functionalized azobenzene derivative with crystallization-induced emission (CIE) or AIEE characteristics associated with the suppression of photoisomerization [54]. Caruso and coworkers synthesized symmetric bis-azobenzene red dyes with relevant fluorescence quantum yield in the solid state [55]. Lee et al. synthesized torsionally responsive *C*_3_-symmetric azo dyes and characterized in detail their reversible conformational switching through twisting of C-N bonds, based on their experimental and computational studies [56].

Nevertheless, there are limited examples of light- and solvent-responsive assembled nano- and microstructures that express both light-driven phase transition and reversible fluorescence on↔off switching functions [57,58,59,60,61,62]. We recently reported that a triangular azo dye designed with consideration of rigidity and flexibility displayed remarkable AIEE and light-induced conformational change from the symmetric to asymmetric structure [60]. However, neither morphological recovery to the original spherical shape nor reversible fluorescence switching was achieved by external stimuli such as light, heat, or pressure.

**Scheme 1:**
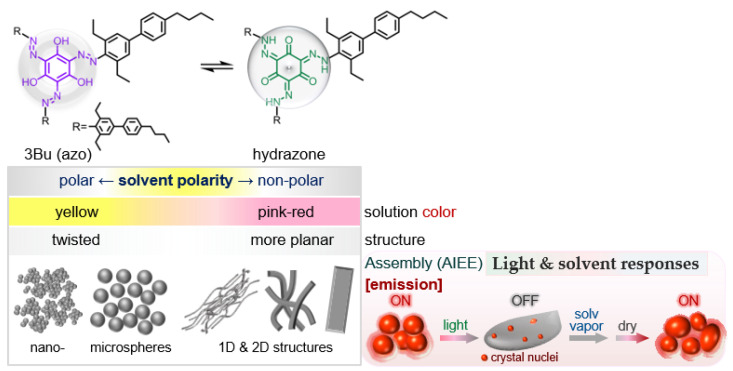
Upper: Chemical structure. Lower left: The plausible molecular structure preferentially taken under different solvent conditions, the resulting solution color, and the morphology of self-assembled structures. Lower right: Light and solvent responses: Subsequent exposure of a light-irradiated sphere sample to solvent vapor resulted in reversible fluorescence off↔on switching accompanied by recovery of the round shape.

Here, we describe the relationship between (i) the plausible conformational and tautomeric structure in which a sterically crowded triangular chromophore (3Bu, Scheme 1) is preferentially adopted depending on the solvent polarity and concentration, (ii) the resulting spectroscopic features, and (iii) the morphology of self-assembled nano-, micro-, and macrostructures formed in various mixed solutions. 3Bu changed the solution color from yellow to pink-red depending on the solvent polarity. A very dilute solution prepared with a more polar solvent such as DMF was yellow, and appeared to contain a significant quantity of a twisted azo form. The highly twisted conformation tended to assemble into less ordered spherical nanoparticles or micrometer-sized spheres showing AIEE characteristics. In contrast, when dissolved in a non-polar solvent such as toluene, the central ring core had a more planar conformation. The resulting keto-hydrazone tautomer grew into micro- and millimeter-sized one-dimensional (1D) structures. Moreover, when a visible-light-irradiated sample was exposed to organic solvent vapors, reverse fluorescence off-to-on switching was realized with recovery of the original morphology.

## 2. Results and Discussion

### 2.1. Solvent Effect

**Figure 1:**
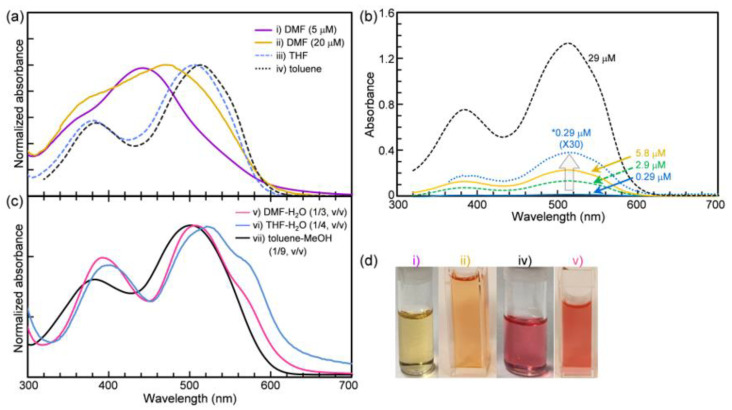
Normalized UV-vis absorption spectra for 3Bu (**a**) in solvents with different polarities and (**c**) in mixed solutions. (**b**) Absorption spectra of 3Bu toluene solutions measured in concentration range from 0.29 to 29 μM. (**d**) Photograph of 3Bu in (i) DMF (5 μM), (ii) DMF (20 μM), (iv) toluene, and (v) DMF-H_2_O mixture. All solutions except for DMF in (**a**) and toluene in (**b**) had a concentration of 10 μM. See Table 1, also.

Sterically crowded 3Bu was thermally stable up to ~210 °C (Figure S1) and dissolved well in common organic solvents such as chloroform, toluene, tetrahydrofuran (THF), and N,N-dimethylformamide (DMF) to make transparent solutions, and the color of the solution observed by the naked eye varied from yellow to pink-red depending on the solvent polarities (Figure 1). A dilute 3Bu DMF solution was yellow, and its UV-vis absorption spectrum contained both a weak shoulder in the region of 330–400 nm and a strong absorption band centered at around 442 nm. This unusual absorption band was presumably due not only to the energetic similarity between the low-lying (π,π*) and (n,π*) states associated with the charge transfer character in the long conjugated system but also to the twisted azobenzene-based n-π* transition [63,64,65,66]. The reason why the molecular central ring connected with three hydroxyl (−OH) groups can adopt a twisted conformation is that three bulky biphenyl wings are directly linked to the central ring via azo (−N=N−) groups. This twisted conformer could possibly be maintained with the help of intermolecular interactions with surrounding polar solvent molecules.

Unlike the absorption spectrum of the yellow DMF solutions, two characteristic absorption bands with maximum values at around 381 and 504 nm appeared in the absorption spectrum for a less polar THF solution. Moreover, upon dissolving in non-polar toluene, the dilute solution showed a bright pink-red color, and two intense absorption bands were red-shifted to 384 and 515 nm, respectively (Figure 1a and Table 1). That is, 3Bu is a negatively solvatochromic compound [26,27,28,29,30,31].

These absorption spectral changes depending on the solvent polarity can be interpreted as follows [67,68,69,70,71]. Non-polar toluene molecules would interact even better with three biphenyl wings than the polar molecular core. It is likely advantageous for the molecular core to adopt a more planar conformation, thereby facilitating intramolecular hydrogen bond formation and proton transfer from the hydroxyl group to the nitrogen atom. The resulting keto-hydrazone form is presumably responsible for the characteristic absorption bands appearing in the long wavelength region above 500 nm. In addition, non-polar toluene solutions in a concentration range from ~0.3 μM to ~30 μM showed almost the same maximum absorption values (Figure 1b).

In contrast to the toluene solution, which was negligibly affected by concentration, the absorption spectra of polar DMF solutions were sensitive to 3Bu concentration. For instance, unlike the quite dilute solution (≤10 μM), a more concentrated solution (≥20 μM) had a maximum absorption band at ~470–480 nm together with a considerably broad shoulder in the 360–450 nm region (Figure 1a,d and Table 1). To further verify gradual molecular structural changes depending on the solvent polarity, absorption spectroscopic measurements were performed by varying the ratio of DMF and toluene at the same concentration of 20 μM (Figure S2). As expected, with increasing toluene content, the broad shoulder in the 360–440 nm region decreased, and instead, two absorption bands maximized at approximately 380 and 510 nm clearly emerged. Notably, as the toluene fraction (*f*_tol_) was increased to 80%, the absorption spectrum was almost the same as that of the sample with *f*_tol_ = 100%. These results suggest that a change in the DMF-toluene mixing ratio created a gradual transition from a twisted to a more planar structure, which in turn promoted the conversion to the keto-hydrazone form.

### 2.2. Theoretical Calculations

**Figure 2:**
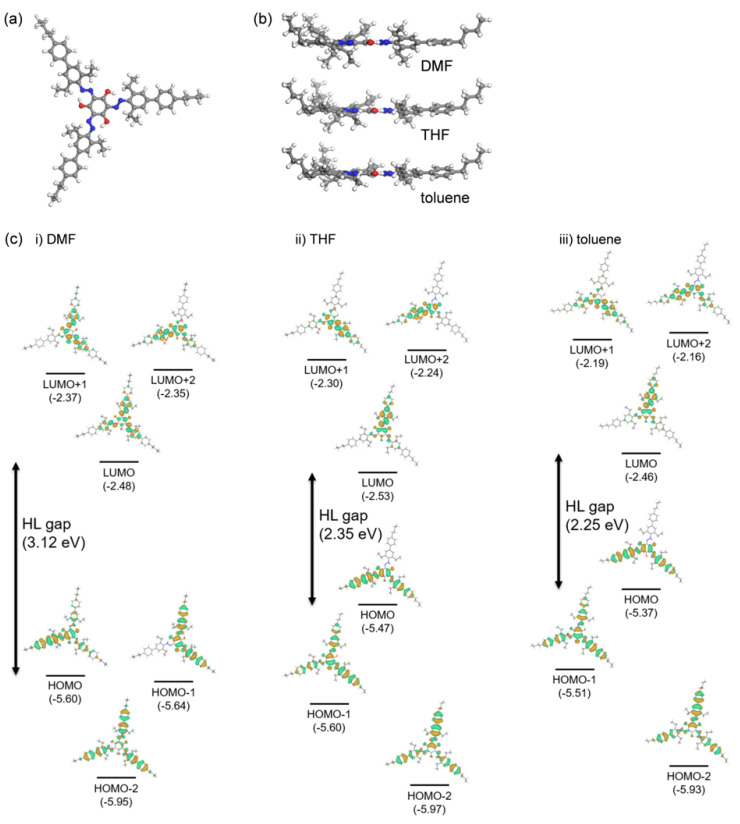
(**a**) Top view of 3Bu molecule optimized in DMF. (**b**) Side views of optimized 3Bu molecules in different solvent media. (**c**) Computationally estimated energy diagram for the frontier molecular orbitals of 3Bu molecule in different solvent media, the influence of which was accounted for using SCRF approximation.

In order to understand the solvatochromism of the 3Bu molecule, density functional theory (DFT) calculations were carried out using a B3LYP functional [72] and 6-31G(d,p) basis set implemented in the Gaussian 09 software package [73]. The influence of a different solvent medium on the geometric and electronic structures was examined using an SMD model within self-consistent reaction field (SCRF) approximation [74]. The most stable geometry had approximately *C*_3_ symmetry regardless of solvent polarity, although the symmetry constraint was not considered during geometry optimizations (Figure 2a). The azo groups were located in the same plane as the central benzene ring due to the intramolecular hydrogen bonding and π-conjugation. The intramolecular hydrogen bonding distances were ~1.56 Å for all solvent mediums. However, the planarity of the 3Bu molecule showed a considerable change depending on the polarity of the solvent medium despite the approximated consideration of the solvent medium (Figure 2b). As the polarity decreased (DMF > THF > toluene), the displacement of ethyl groups from the molecular plane increased, and they were attached to the first benzene ring of the side group. We examined the variation of molecular planarity with the torsional angle (θ_1_) between the azo group and the first benzene ring of a side chain and identified the orientation of the side group with three torsional angles (inset figure in Table 2), while the torsional angles θ_2_ and θ_3_ were almost identical for all solvent mediums, and the torsional angle θ_1_ gradually decreased (i.e., the planarity increased) as the solvent polarity decreased (Table 2). The frontier molecular orbitals, mainly characterized as π-orbitals, were dominantly distributed from the central benzene ring to the first benzene ring of the side group (Figure 2c). Therefore, the energy gap between the highest occupied and the lowest unoccupied molecular orbitals (HOMO-LUMO gap) was highly influenced by the variation of the torsional angle θ_1_, and thus a larger HOMO-LUMO gap was obtained for a more highly polar solvent (Table 2), which agrees quite well with the experimental observation (see Figure 1). Time-dependent DFT (TD-DFT) calculations were further performed to reveal the solvatochromism of the 3Bu molecule. The computationally obtained absorption wavelength (λ_max_ for S_1_←S_0_) was also roughly comparable to the HOMO-LUMO gap and experimental results (Table 2).

### 2.3. Self-Assembly into Spherical and 1D Structures

**Figure 3:**
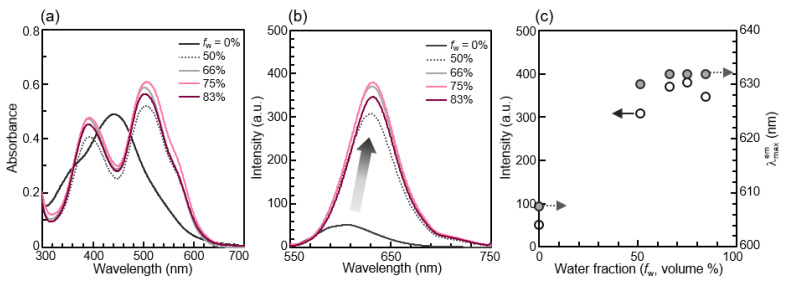
(**a**) Absorption and (**b**) fluorescence (λ_ex_= 510 nm) spectra of 3Bu in DMF-H_2_O mixed solutions (10 μM) prepared by varying the water fraction (*f*_w_). (**c**) Changes in the fluorescence intensity at the maximum fluorescence wavelength as a function of *f*_w_.

**Figure 4:**
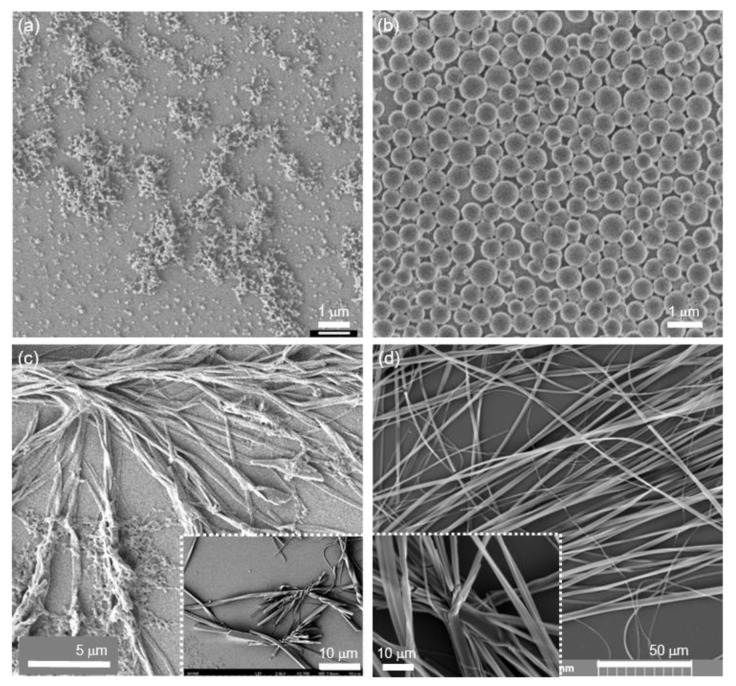
SEM images of self-assembled structures obtained from (**a**) DMF-H_2_O (1/3, *v*/*v*, 10 μM), (**b**) THF-H_2_O (1/4, *v*/*v*, 20 μM), (**c**) DMF-H_2_O (1/1, *v*/*v*, 100 μM), and (**d**) toluene-MeOH (1/4, *v*/*v*, 100 μM) mixtures.

To investigate how such delicate changes in conformational or tautomeric structures affect their molecular assemblies and the resulting spectroscopic features, we next produced self-assembled nano- and microstructures using a reprecipitation method. Figure 3 shows the absorption and fluorescence spectral changes of 3Bu DMF-H_2_O mixtures prepared by varying the water fraction while keeping the concentrations at 10 μM. As soon as a few drops of ultrapure water, which is a poor solvent, were added into a 3Bu DMF solution under gentle shaking, the color of the mixed solution dramatically changed from yellow to bright red, which could be clearly observed with the naked eye (inset photo in Figure 1d). The characteristic absorption band at ~442 nm disappeared completely, and two intense absorption bands at ~392 and 505 nm emerged (Figure 3a). At the same time, the maximum fluorescence wavelength was red-shifted from 608 nm to 630 nm, and the intensity increased considerably (Figure 3b,c). As the water fraction was increased to 75%, the fluorescence intensity reached the maximum, which was about 7.5 times higher than that of the dilute DMF solution. These results support that 3Bu is AIEE-active [47,48,49,50,51,52]. Our careful scanning electron microscopy (SEM) observations revealed the formation of nanometer-sized spherical particles with diameters of ~10–100 nm (Figure 4a). Such small aggregates were often found as agglomerates rather than being dispersed on the hydrophilic glass substrate. In addition, a turbid suspension produced by adding H_2_O to a less polar THF solution displayed absorption maxima at 400 and 517 nm (Figure 1c). Micrometer-sized spheres with sizes of ~0.1–1.0 µm were found in the concentration range of 1.0–25 µM (Figure 4b).

Unlike the dilute DMF-H_2_O mixtures, mixed solutions with concentrations of 50 µM or higher did not become turbid, but rather, insoluble floats were generated and gradually sank to the bottom of a quartz cuvette with a 10 mm path length. The SEM images presented in Figure 4c verify the coexistence of long sticks and intertwined fibers with length of tens of micrometers instead of spheres. In other words, the morphological transformation from nanoparticles to micrometer-sized 1D structures was realized simply by varying the concentration.

In toluene-MeOH mixed solutions, cotton-like precipitates were very slowly produced over 1 to 3 weeks without becoming turbid and slowly separated from the transparent solution. Figure 4d and Figure S3 show that such fluffy precipitates were composed of both long sticks hundreds of micrometers in length and elongated fibers that were several millimeters long. The respective 1D structures were red fluorescent upon excitation with green light (Figure S3b). As evidenced by our SEM observations, the fibrous structures appeared to be flexible and were often broken into two or more pieces, suggesting that they were crystalline.

### 2.4. Coexistence of Spherical, 1D and 2D Structures

**Figure 5:**
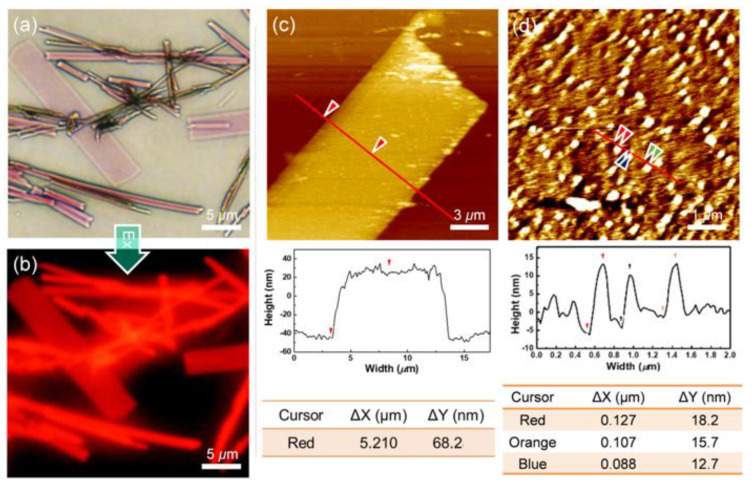
(**a**) OM and (**b**) FOM images of 3Bu belts and sheets obtained from 25 μM THF-H_2_O (1/1, *v*/*v*) at 10–15 °C. (**c**) AFM image showing 3Bu microsheet on a glass substrate and height profile. (**d**) Magnified AFM image showing spherical nanoparticles on 3Bu microsheet and height profile.

**Figure 6:**
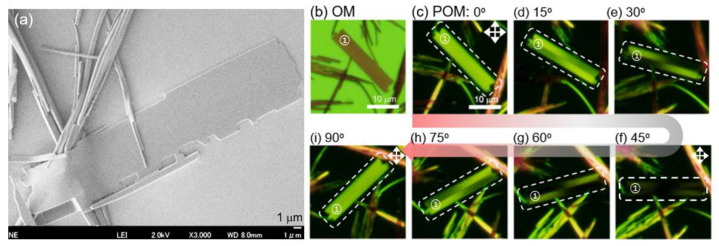
(**a**) SEM image of belts and sheets prepared from 25 μM THF-H_2_O (1/1, *v*/*v*) at 10–15 °C. (**b**) OM and (**c**–**i**) POM images of birefringent microsheets placed between the crossed polarizers. The sample direction was rotated by 15 degrees with respect to the crossed polarizers.

Surprisingly, in the case of a 25 μM THF-H_2_O (1/1, *v*/*v*) mixed solution, when the turbid mixture was stored at 10–15 °C, unlike other ratios in which turbid mixed solutions remained stable, visible floats were produced. They gradually sank to the bottom of the quartz cell, and as a result, the solution became transparent. The OM and FOM images of the precipitates reveal that light purple rectangular aggregates with red fluorescence coexisted with long, flat belts of dark purple color (Figure 5a,b). Uneven ends and corners were frequently found in the OM, atomic force microscopy (AFM), and SEM images (Figure 5 and Figure 6), and the samples may have been torn during growth or during their preparation. Their thickness and width were approximately 60–100 nm and 1–20 μm, respectively. AFM topographic images obtained for sheets exhibited uneven surfaces regardless of their size (Figure 5c,d). Very tiny spherical nanoparticles of approximately 10–20 nm in height were frequently observed on such sheet surfaces (Figure 5d). Notably, from polarized optical microscopy (POM) images measured under crossed Nicol conditions, when the long axis of the rectangular sheet was placed at approximately 45° (Figure 6c,i) and 0° (Figure 6f) with respect to both the polarizer and analyzer, it was observed to be maximally bright and maximally dark, respectively [75,76,77,78]. These data support that the triangular molecules were well aligned in the long axis direction (in-plane alignment) to form anisotropic sheets.

**Figure 7:**
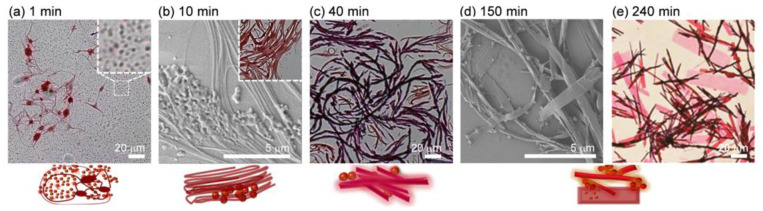
OM (**a**, inset image in **b**,**c**,**e**) and SEM (**b**,**d**) images showing the morphological growth processes of 3Bu THF-H_2_O (1/1, *v*/*v*) mixtures according to storage time at 10–15 °C.

Additional OM and SEM observations were carried out to obtain insight into the growth process of fluorescent anisotropic sheets. Upon addition of water to the THF solution, purple agglomerates of approximately 20 μm or less, interconnected by elongated fibers, were frequently found along with spherical particles with a diameter of approximately 500 nm or less (Figure 7a). As time passed, these fibers began to pile up sideways, reaching a width of several hundred nanometers to several micrometers (Figure 7b and Figure S4a). The OM and SEM images in Figure 7c and Figure S4b show that they grew into branches and belts. After about 2–4 h, they developed into micrometer-sized sheets, and gradually sank to the bottom along with long belts (Figure 7d–e). The growth process of the 2D structures through the lateral stacking of 1D fibers is likely responsible for the strong birefringence of micrometer-sized sheets. Although it is still unknown why 1D fibers prefer such lateral stacking, low temperature and the mixing ratio of the solvents appear to be important factors in developing spherical and 1D structures into fluorescent 2D sheets.

### 2.5. FT-IR and XRD Measurements

**Figure 8:**
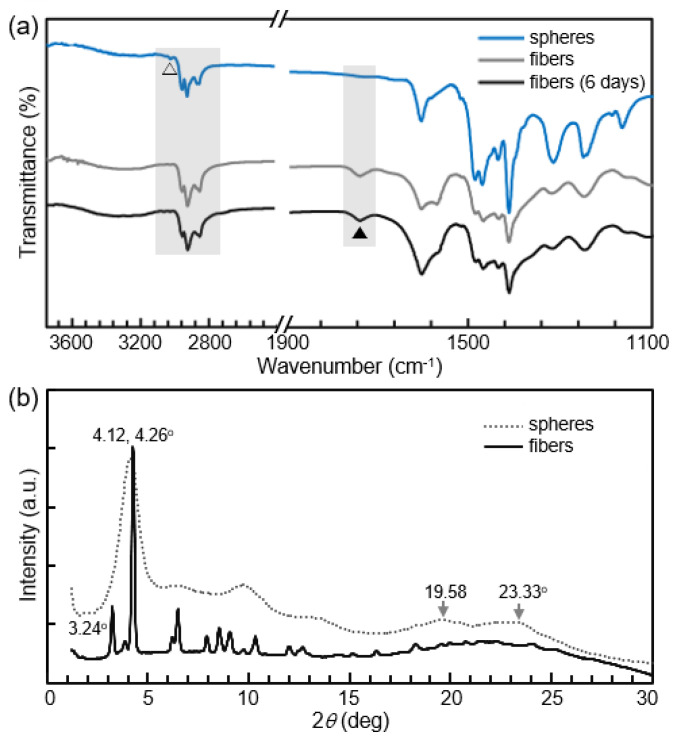
(**a**) FT-IR and (**b**) XRD patterns of spherical and 1D fibrous structures in (**a**), “6 days” denotes a sample dried in a vacuum for 6 days.

Fourier-transform infrared (FT-IR) and X-ray diffraction (XRD) experiments were performed to obtain useful information on (i) the molecular structures and (ii) the degree of molecular arrangement responsible for the respective spherical and 1D assembles. Importantly, a weak band maximized at around 1730 cm^−1^ and a quite broad band at 3100–3500 cm^−1^ emerged in the IR spectrum of intertwined fiber samples produced from toluene-MeOH mixtures (Figure 8a), and they could be attributed to carbonyl stretching and hydrogen bonding, respectively [79,80,81]. Rather than a typical ketone, the carbonyl is considered to experience ring strain or to be close to an electron-withdrawing substituent [79,80]. For confirmation, we reproduced such 1D structures in the same manner and dried them in a vacuum for 6 days to sufficiently remove residual solvents. The obtained sample also exhibited the same absorption band. These results indicate that a considerable amount of the hydrazone form present in a toluene solution was retained even after fiber formation.

By contrast, the IR spectrum of the spherical aggregates displayed an aromatic C-H stretching vibration at around 3011 cm^−1^ and aromatic C=C stretching bands at 1601 and 1484 cm^−1^, but did not show any absorption bands corresponding to the carbonyl stretching mode. It is thus reasonable to interpret that a significant amount of 3Bu, which existed in the twisted azo form rather than in the hydrazone form, tended to assemble quickly into less ordered spherical structures.

In contrast to spheres showing soft and weak signals, sharp and higher-intensity peaks were recorded in the XRD pattern for the crystalline fibrous structures (Figure 8b), as predicted earlier. The sharp and strongest signal appeared near 2*θ* = 4.26° (d = 2.08 nm), which is slightly shorter than the full molecular length (2.15 nm) predicted by the theoretical calculation. On the other hand, the sphere sample showed almost the same XRD pattern as in the previous report [60], and had a strong peak at 2*θ* = 4.12° (d = 2.14 nm) corresponding to the molecular length. Fairly weak peaks appeared at 2*θ* = 19.58 (d = 0.45 nm) and 23.33° (d = 0.38 nm), reflecting that intermolecular distances were longer than those of typical π–π stacking interactions between planar aromatic units [82,83,84,85]. In addition, such broad peaks were rarely observed in the XRD pattern of crystalline fiber samples. This can be interpreted as follows. The triangular 3Bu molecule with three bulky *ortho*-alkylated biphenyl wings is highly sterically crowded [63,64,65,66,86,87,88,89], and intramolecular rotation around a single or double bond is greatly restricted. As a result, considerable occupied space per molecule is secured, and intermolecular interactions are weakened. For this reason, although dense packing is difficult, it appears that conditions for the molecules to grow into spherical and one-dimensional structures in the mixed solutions were provided.

### 2.6. Fluorescence Switching Accompanied by Morphological Transition

**Figure 9:**
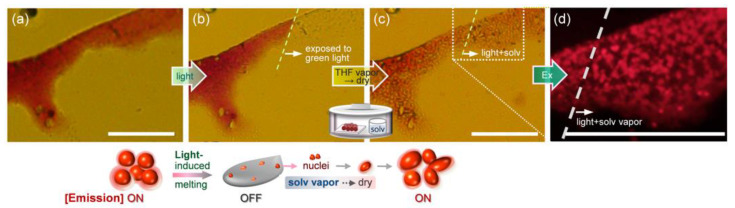
Reversible fluorescence switching: When spherical aggregates on a quartz substrate were exposed to (**a**,**b**) visible (green) light, a significant color change (due to light-induced melting) occurred with a reduction in the fluorescence intensity. (**c**) OM and corresponding (**d**) magnified FOM images taken after exposure of the sphere sample to THF vapor for 3 hours. Their morphological recovery caused the sample to fluoresce again. Scale bar: 50 μm.

To realize reversible fluorescence switching of less ordered spheres with AIEE characteristics, between on and off states, we devised an experimental method to utilize the result that the visible light-induced sphere-to-liquid phase transition is accompanied by fluorescence on-to-off switching (the fluorescence quantum yield (Φ_f_) decreased from 2.4% to ~0.4%). We assumed that, if a light-irradiated sample is exposed to solvent vapor, the movement of 3Bu molecules becomes more random due to intermolecular interactions between chromophore and solvent molecules. However, if the molecular arrangements in the spherical aggregates could be maintained to some extent, because the spatial arrangements of the 3Bu molecules could be amplified during the drying process after exposure to solvent vapor, the original shape could be restored to some extent. To test this assumption, we exposed a green-light-irradiated sphere sample to THF vapor (with a low boiling temperature) in a sealed bottle (Figure 9). When the sample was removed from the bottle after 3 hours and slowly dried at ambient temperature, the original round shape was restored. At the same time, the fluorescence intensity also improved (Φ_f_ = ~1.9%). However, if the sample was exposed to solvent vapor for too long (Figure S5) and the spatial arrangements disappeared completely, it was almost impossible to achieve sufficient morphological recovery.

## 3. Conclusions

We investigated the relationship between (i) the conformational and tautomeric structure in which a triangular chromophore preferentially is adopted depending on the solvent polarity and the concentration, (ii) the resulting spectroscopic features, and (iii) self-assembled nano-, micro-, and macrostructures formed in various mixed solutions. The combination of our experimental results and theoretical calculations revealed that, in a very dilute DMF solution, the triangular molecule present in a highly twisted azo form tended to assemble into less ordered spherical nanoparticles or microspheres. In contrast, in non-polar toluene solutions, the polar central ring part adopted a more planar form, thereby promoting conversion to the hydrazone form. The resulting tautomer grew slowly into crystalline micro- and millimeter-sized 1D structures. Interestingly, upon storing the THF-H_2_O mixtures at a low temperature, birefringent 2D sheets were created through the lateral stacking of elongated fibers. Furthermore, the shape and fluorescence intensity of sphere samples could change in response to stimuli such as light and solvent vapor. These findings may be useful for developing new fluorescent materials for chemical detection as well as for rewritable data storage.

## 4. Materials and Methods

### 4.1. Materials

Spectroscopic grade organic solvents (N,N-dimethylformamide (DMF], tetrahydrofuran (THF], toluene, methanol (MeOH), chloroform) were purchased from Kanto Chemical Co., INC, Japan. Ultrapure water (which was purified to reach a minimum resistivity of 18.0 MΩ·cm (25 °C) using a μPure HIQ water purification system, Romax, South Korea) was utilized in all experiments.

### 4.2. Characterization

After a 30 s nitrogen purge, a screw-cap quartz cuvette containing 3Bu solution was sealed with Parafilm^®^. UV-vis absorption and fluorescence spectra were obtained using a Shimadzu UV-2600 UV-vis spectrophotometer (Shimadzu Corporation, Kyoto, Japan) and a Horiba FluoroMax-4 spectrofluorometer (Horiba Ltd., Japan), respectively. Optical microscopy (OM) and fluorescence optical microscopy (FOM, λ_ex_ = 520–550 nm) experiments were performed using an Olympus BX53 microscope after placing two to three drops of 3Bu mixed suspension onto a clean glass or quartz substrate. Polarized optical microscopy (POM) images were obtained using an Olympus BX53-P polarizing microscope (Olympus Corporation, Tokyo, Japan). Atomic force microscopy (AFM) measurements were performed using XE-100 (PSIA, Corp., Korea) equipped with a 50 μm scanner. FE-SEM (field-emission scanning electron microscopy: HITACHI SU8020 (Hitachi High-Technologies Corporation, Tokyo, Japan) and TESKAN-MIRA3-LM (TESCAN ORSAY HOLDING, a.s., Czech Republic) samples were coated with an approximately 5–10 nm thick platinum layer using a Cressington 108 auto sputter coater (Ted Pella, Inc., CA, USA) The thermal stability of 3Bu was determined by a thermogravimetric analysis (TGA) method using a Pyris 1TGA Thermogravimetric Analyzer (PerkinElmer, Inc., Waltham, MA, USA). All measurements were performed under a nitrogen atmosphere in a temperature range of 22 to 490 °C. All experiments were performed twice at a heating rate of 5 °C/min. Fourier transform infrared (FT-IR) spectra were recorded on a PerkinElmer (spectrum 100) spectrometer. X-ray diffraction (XRD) data were collected with Cu Kα radiation on Rigaku R-AXIS-IV and R-AXIS-VII X-ray imaging plate detectors to determine the structure of the 3Bu molecule.

## Figures and Tables

**Table 1:** Solvent properties, spectroscopic data, and self-assembled structures of 3Bu obtained from various solvent environments.

Solvent	DMF	DMF-H_2_O	THF	THF-H_2_O	Toluene	Toluene-MeOH
polarity	13.7	-	5.7	-	1.4	-
dielectric constant	38.2	-	7.5	-	2.4	-
$\lambda_{max}^{abs}$	442→470–480	392, 505	381, 504	400, 517	384, 515	383, 502
$\lambda_{max}^{em}$	608	632	610	629	616	617
aggregate morphology	-	nanoparticle→1D structures	-	sphere (1D→2D structures)	-	1D structures
AIEE property	-	○	-	○	-	○

**Table 2:** Computationally obtained average dihedral angles, HOMO-LUMO gaps, and absorption wavelengths depending on solvent medium. Inset: Two-dimensional structural skeleton of 3Bu, where intramolecular hydrogen bonds are red-colored and three torsional angles are blue-colored.

Solvent		DMF	THF	Toluene
Average dihedral angle (°)				
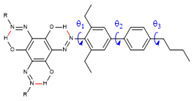	θ_1_	27.1	18.5	13.3
	θ_2_	−34.2	−34.8	−35.4
	θ_3_	89.7	89.7	88.9
HOMO-LUMO gap (eV)		3.12	2.35	2.25
Abs. wavelength (λ_max_, nm)		473.9	491.8	497.2

## Data Availability

The data are included within the manuscript and Supplementary Materials.

## Supplementary Materials

The following supporting information can be downloaded at: https://www.mdpi.com/article/10.3390/ijms23020965/s1.
