# Supplementary material for: Solvent- and Light-Sensitive AIEE-Active Azo Dye: From Spherical to 1D and 2D Assemblies
Source: Int J Mol Sci. 2022 Jan 16;23(2):965. doi: 10.3390/ijms23020965 (PMC8778914; doi:10.3390/ijms23020965)
Supplement: Supplementary file 1 [file ijms-23-00965-s001.zip › ijms-1542467-supplementary.pdf]

Supplementary Materials (SM)

Solvent- and Light-Sensitive AIEE-Active Azo Dye: From Spherical to 1D and 2D Assemblies

Mina Han, Ikue Abe, Jihun Oh, Jaehoon Jung, Young Ji Son, Jaegeun Noh, Mitsuo Hara and Takahiro Seki

Contents

Figure S1. TGA curve of 3Bu under nitrogen atmosphere.

Figure S2. UV-vis absorption spectral changes of 3Bu in toluene-DMF mixtures (20 μM) with different volume ratios.

Figure S3. (a) OM and corresponding (b) FOM (λ_{ex} = 520−550 nm) images of 1D structures obtained from toluene-MeOH mixture (1:4, v/v, 20 μM).

Figure S4. SEM images showing the morphological growth processes of 3Bu THF-H_2O (1/1, v/v, 25 μM) mixtures according to storage time at 10-15°C.

Figure S5. OM images taken (a) before and (b) after exposure of a fiber sample to THF vapor for 8 hours.

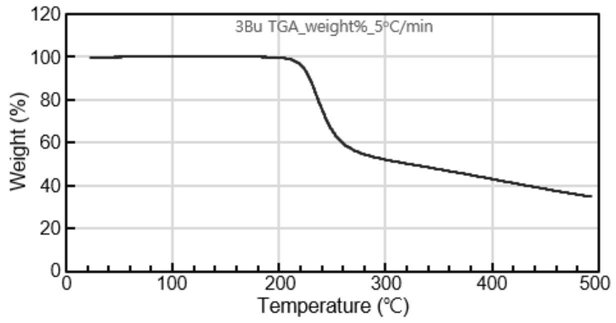

Figure S1. TGA curve of 3Bu under nitrogen atmosphere.

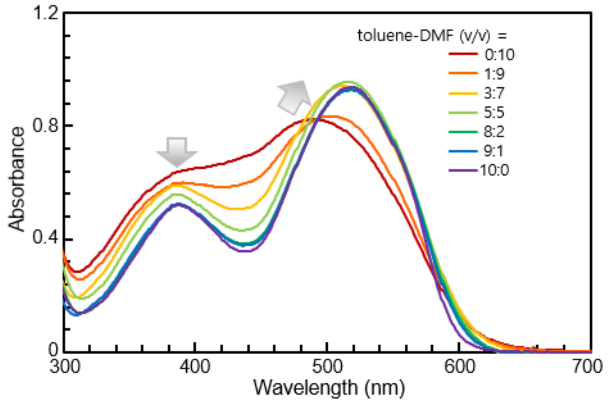

Figure S2. UV-vis absorption spectral changes of 3Bu in toluene-DMF mixtures (20 μM) with different volume ratios.

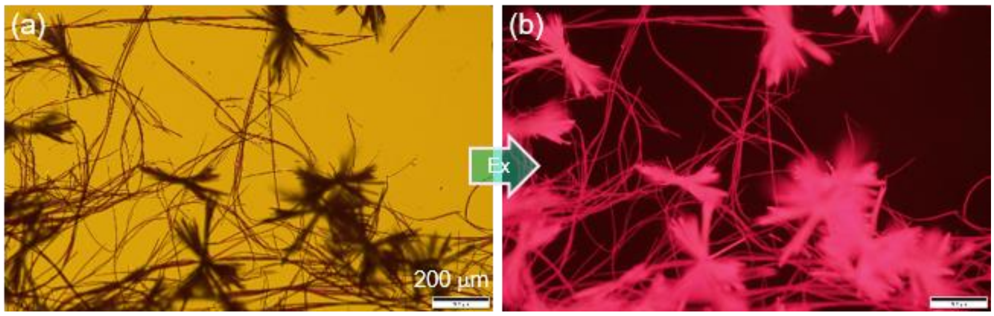

Figure S3. (a) OM and corresponding (b) FOM (λ_{ex} = 520–550 nm) images of 1D structures obtained from toluene-MeOH mixture (1:4, v/v, 20 μM).

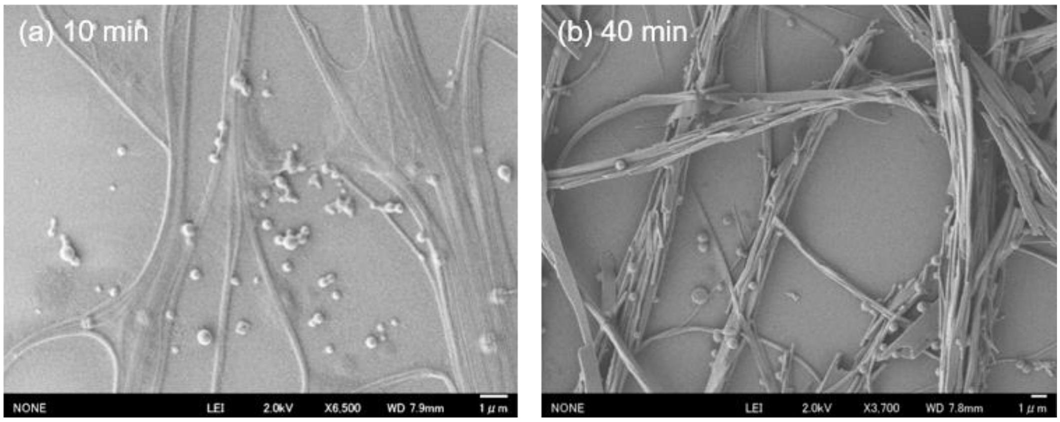

Figure S4. SEM images showing the morphological growth processes of 3Bu THF-H_2O (1/1, v/v, 25 μM) mixtures according to storage time at 10-15°C.

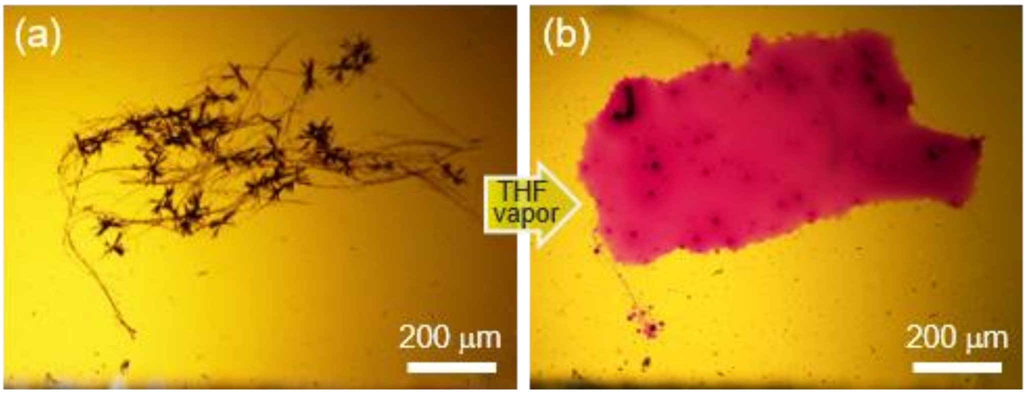

Figure S5. OM images taken (a) before and (b) after exposure of a fiber sample to THF vapor for 8 hours.
